# Sociodemographic Differences in Prenatal Diagnosis of Chromosomal Anomalies: A Population-Based Study

**DOI:** 10.3389/fped.2021.630363

**Published:** 2021-02-04

**Authors:** Michele Santoro, Lorena Mezzasalma, Alessio Coi, Silvia Baldacci, Lucia Pasquini, Anna Pierini

**Affiliations:** ^1^Unit of Epidemiology of Rare Diseases and Congenital Anomalies, Institute of Clinical Physiology, National Council of Research, Pisa, Italy; ^2^Fetal Medicine Unit, Department for Women and Children Health, Azienda Ospedaliero-Universitaria Careggi, Florence, Italy; ^3^Fondazione Toscana Gabriele Monasterio, Pisa, Italy

**Keywords:** chromosomal anomalies, down syndrome, prenatal diagnosis, maternal education, maternal geographic origin, maternal occupation, socioeconomic status

## Abstract

**Background:** In Europe, about 76% of cases of chromosomal anomalies are prenatally diagnosed. Prenatal diagnosis allows more efficient planning of postnatal treatment and helps parents for an informed decision about the continuation of pregnancy. The main aim of this study was to evaluate whether the sociodemographic maternal characteristics affect the probability of prenatal diagnosis of chromosomal anomalies.

**Methods:** Cases of chromosomal anomalies in the period 2005–2017 came from the population-based registry of congenital anomalies of Tuscany (Italy). Differences in the proportion of cases prenatally diagnosed were investigated through the following maternal characteristics: education, geographic origin and occupation. The association between cases of termination of pregnancy after prenatal diagnosis and maternal characteristics was also analysed. Odds Ratios (OR) adjusted by maternal age were calculated using logistic regression models. Results were provided for all cases of chromosomal anomalies and for Down syndrome cases.

**Results:** A total of 1,419 cases were included in the study. Cases prenatally diagnosed were 1,186 (83.6%). We observed a higher proportion of cases not prenatally diagnosed among cases with low maternal education compared to those with high maternal education (OR = 2.16, *p* < 0.001) and in women from high migratory outflow countries, compared to the Italian ones (OR = 2.85, *p* < 0.001). For prenatally diagnosed Down syndrome cases, we observed a higher proportion of termination of pregnancy for women with low education level (OR = 4.36, *p* = 0.023).

**Conclusions:** In our study evidence of differences in the probability of prenatal diagnosis of chromosomal anomalies associated with maternal education and geographic origin was found. Population-based studies investigating sociodemographic disparities can provide essential information for targeted public health programs. Further studies are recommended to monitor the impact of the increasing availability of non-invasive screening tests.

## Introduction

Chromosomal anomalies account for about 17% of the major congenital anomalies diagnosed before the age of 1 year in Europe. According to EUROCAT, the European network of population-based registries for the epidemiological surveillance of congenital anomalies, the proportion of chromosomal anomalies prenatally diagnosed is around 76% (1). Prenatal diagnosis may allow more efficient planning of postnatal medical and surgical care of the neonate born with a congenital anomaly. Moreover, prenatal diagnosis constitutes support for parents for an informed decision about the continuation of pregnancy.

The effects of socioeconomic factors on the prevalence and the access to prenatal diagnosis of Down syndrome, the most frequent chromosomal anomaly, have been investigated in a few population-based studies (2–5). The results of these studies suggest the presence of disparities in prenatal diagnosis of Down syndrome among the different ethnic and socioeconomic subgroups. Moreover, differences in the access to prenatal diagnosis of sociodemographic subgroups generate differences in live-birth prevalence across the same subgroups (4).

The Italian healthcare system is structured with two different levels of responsibility and management: a central level and a regional level. The former ensures and guarantees universal access to essential services, regardless of the socioeconomic status (SES); the latter has direct responsibility for management and costs with exclusive competence for the regulation and organisation of services, including those during pregnancy and at birth. However, SES-based disparities persist and affect several dimensions of general health, including access to prenatal diagnosis. In Italy, despite active policies aimed at increasing prenatal testing, little is known (6, 7) about the impact of social changes, both economic and demographic, that have taken place in the last decades.

To meet the needs of this investigation, maternal education, a recognised powerful determinant of health (8), was chosen as a proxy of SES. Since the foreign presence on the national territory is a consolidated phenomenon, the maternal geographic origin was studied as another possible determinant of the access to prenatal diagnosis (9–12). Furthermore, we also explored the possible association with the maternal occupation.

Thus, the main aim of the present study was to evaluate whether the sociodemographic maternal characteristics have a role in the probability of prenatal diagnosis of chromosomal anomalies. Furthermore, we investigated whether these factors were associated with the proportion of termination of pregnancy for foetal anomaly following prenatal diagnosis (TOPFA).

## Materials and Methods

The study population consisted of all cases with chromosomal anomalies (live births, foetal deaths with gestational age ≥20 weeks and TOPFA) diagnosed during the first year of life for women residing in Tuscany (Italy) in the period 2005–2017. Tuscany is an Italian region with about 3,700,000 inhabitants and 25,000 births yearly. Data on cases of chromosomal anomalies came from the population-based Registry of Congenital Defects of Tuscany (RTDC, from the Italian acronym of “Registro Toscano dei Difetti Congeniti”). RTDC is a full member of EUROCAT (13) and the International Clearinghouse for Birth Defects Surveillance and Research (ICBDSR) (14). The case collection is based on a widespread network, including all obstetrical and maternity units, paediatric departments, paediatric cardiology departments, paediatric cardiac surgery units, prenatal diagnostic centres, and medical genetics units, which ensures complete geographical coverage. Cases from RTDC are linked with other regional health databases.

Differences in the probability of prenatal diagnosis for chromosomal anomalies were investigated through the following maternal characteristics: education, occupation, and geographic origin. Maternal education was stratified into three levels: low (primary and lower secondary level), medium (upper secondary level), high (tertiary level). Data on maternal education came from RTDC and the regional birth registry. Data on maternal occupation were collected in RTDC using the International Standard Classification of Occupations (ISCO-88 for births up to 2012, ISCO-08 for births from 2013). For our study, we have slightly modified the classification adopted by Zeitlin et al. (15) and we have defined the following 4 groups: manager/professional, technical/clerical/service, unskilled occupation/unemployed, housewives. The geographic origin of the mother was derived from the integrated information reported in the RTDC and the birth registry. The country of origin was classified as Italy, Developed Countries (DC) other than Italy, and High Migratory Outflow Countries (HMOC) (16). Among HMOC the most represented communities in Tuscany are from Romania, Albania, followed by China and Morocco (17). Maternal age was categorised into 3 classes: <35 years, 35–39 years, and 40+ years.

Logistic regression models, adjusted by maternal age, were used to estimate the association of maternal education, occupation, and geographic origin with prenatal diagnosis. For maternal education and occupation, estimates adjusted for geographic origin were also calculated. Adjusted Odds ratios (OR) with 95% confidence interval (95% CI) and *p*-value were calculated. Analyses were performed for all cases with chromosomal anomalies, for cases with Down syndrome only, and cases with a major trisomy. The association between TOPFA and maternal characteristics was analysed in prenatally diagnosed cases using a logistic regression model. The difference in gestational age at diagnosis was evaluated using the Wilcoxon test. Analyses stratified by period (2005–2011 vs. 2012–2017) were also performed. Results with a *p* < 0.05 were defined as statistically significant. Statistical analyses were performed using STATA version 16.0 (StataCorp LP, College Station, TX, USA).

## Results

A total of 1,419 cases with chromosomal anomalies were included in the study. Out of them, 838 were cases of Down syndrome (59.1%). The other more frequent chromosomal anomalies were trisomy 18 (15.2%), trisomy 13 (4.5%), Turner syndrome (5.9%), and Klinefelter syndrome (4.1%). Overall, prenatally diagnosed cases were 1,186 (83.6%). Among them, 122 were liveborn. The proportion of cases with low maternal education was 22.6%. About 18% of the mothers were unemployed or with an unskilled occupation. Mothers from HMOC were 17.0% and from not Italian DC 2.0%. About 30% of the mothers were aged 40 years and older (Table 1). Maternal age was strongly associated with prenatal diagnosis (*p* < 0.001).

**Table 1:** Characteristics of the study population.

**Maternal characteristics**	**All chromosomal anomalies**		**Down syndrome**	
	***n*. (%)**	**Missing**	***n*. (%)**	**Missing**
**Prenatal diagnosis**				
Yes	1186 (83.6)		651 (77.7)	
No	233 (16.4)		187 (22.3)	
Missing		–		–
**Age**				
<35	427 (30.3)		212 (25.5)	
35–39	557 (39.5)		343 (41.2)	
≥40	426 (30.2)		277 (33.3)	
Missing		9 (0.6)		6 (0.7)
**Education**				
High	277 (30.2)		166 (29.8)	
Medium	432 (47.2)		260 (46.7)	
Low	207 (22.6)		131 (23.5)	
Missing		503 (35.5)		281 (33.5)
**Occupation**				
Manager/Professional	118 (14.9)		69 (14.3)	
Technical/Clerical/Service	495 (62.4)		300 (62.2)	
Unskilled occupation/	142 (17.9)		92 (19.1)	
Unemployed				
Housewives	38 (4.8)		21 (4.4)	
Missing		626 (44.1)		356 (42.5)
**Geographic origin**				
Italian	1032 (80.7)		619 (81.5)	
DC	29 (2.3)		19 (2.5)	
HMOC	217 (17.0)		122 (16.0)	
Missing		141 (9.9)		78 (9.3)
**Study period**				
2005–2011	730 (51.4)		444 (53.0)	
2012–2017	689 (48.6)		394 (47.0)	
Missing		–		–

*DC, Developed Countries; HMOC, High Migration Outflow Countries*.

We observed a higher risk of not having a prenatal diagnosis in cases with low maternal education compared to high maternal education (OR = 2.16, *p* < 0.001) (Table 2). The proportion of cases without a prenatal diagnosis was higher also when adjusted for maternal geographic origin (OR = 1.89, *p* = 0.01).

**Table 2:** Adjusted Odds Ratio of not having a prenatal diagnosis by maternal characteristics for all chromosomal anomalies.

	**OR^#^**	**95% CI**	***P***	**OR^§^**	**95% CI**	***P***
**Education**						
High	Ref.			Ref.		
Intermediate	1.07	0.72–1.59	0.728	1.05	0.68–1.63	0.811
Low	2.16	1.41–3.32	0.000	1.89	1.16–3.09	0.010
**Occupation**						
Manager/Professional	Ref.			Ref.		
Technical/Clerical/Service	0.68	0.36–1.27	0.229	0.64	0.34–1.20	0.163
Unskilled occupation/	1.96	1.00–3.84	0.051	1.32	0.65–2.70	0.445
Unemployed						
Housewives	0.72	0.22–2.34	0.583	0.46	0.13–1.55	0.210
**Geographic origin**						
Italian	Ref.					
DC	2.68	1.11–6.45	0.028			
HMOC	2.85	2.00–4.07	0.000			

*OR, Odds Ratio*.

# *Adjusted by maternal age*.

§ *Adjusted by maternal age and geographic origin*.

*DC, Developed Countries; HMOC, High Migration Outflow Countries*.

Regarding maternal occupation, we observed a higher proportion of not prenatally diagnosed cases, borderline significant, in the group “unskilled occupation/unemployed” (OR = 1.96, *p* = 0.051). When adjusted by geographic origin the risk was no more significant.

Mothers from HMOC and mothers from DC other than Italy, compared to Italian ones, had a higher proportion of not prenatally diagnosed cases (OR = 2.85, *p* < 0.001 and OR = 2.68, *p* = 0.028, respectively). These results were also confirmed when adjusted by maternal education (OR = 2.96, *p* < 0.001 and OR = 2.94, *p* = 0.03, respectively).

Among all the cases of Down syndrome, 77.7% were prenatally diagnosed. As reported in Table 3, a higher risk of not having a prenatal diagnosis was observed in mothers with low education (OR = 2.23, *p* = 0.002), also when adjusted by geographic origin (OR = 1.79, *p* = 0.047).

**Table 3:** Adjusted Odds Ratio of not having a prenatal diagnosis by maternal characteristics in Down syndrome.

	**OR^#^**	**95% CI**	***P***	**OR^§^**	**95% CI**	***P***
**Education**						
High	Ref.			Ref.		
Intermediate	1.06	0.67–1.67	0.807	0.99	0.60–1.64	0.963
Low	2.23	1.34–3.71	0.002	1.79	1.01–3.16	0.047
**Occupation**						
Manager/Professional	Ref.			Ref.		
Tech/Clerical/Service	0.47	0.24–0.93	0.029	0.39	0.19–0.78	0.008
Unskilled occupation/	1.53	0.73–3.21	0.258	1.03	0.47–2.26	0.946
Unemployed						
Housewives	0.70	0.20–2.47	0.574	0.43	0.11–1.62	0.213
**Geographic origin**						
Italian	Ref.					
DC	2.79	1.02–7.65	0.045			
HMOC	3.73	2.43–5.73	0.000			

*OR, Odds Ratio*.

# *Adjusted by maternal age*.

§ *Adjusted by maternal age and geographic origin*.

*DC, Developed Countries; HMOC, High Migration Outflow Countries*.

Concerning maternal occupation, no significantly higher risk was observed in the group “unskilled occupation/unemployed,” whereas a lower proportion of cases was observed in the group “technical/clerical/ service” (OR = 0.47, *p* = 0.029).

Mothers from HMOC and DC had a higher risk than Italian mothers of not having a prenatal diagnosis (OR = 3.73, *p* < 0.001 and OR = 2.79, *p* = 0.045, respectively).

Looking at all the major trisomies (i.e. trisomies 13, 18 and 21), the overall number of cases was 1,118, and 82.4% were prenatally diagnosed. The associations with maternal risk factors were confirmed with a higher proportion of not prenatally diagnosed cases in mothers with low education level and mothers from HMOC.

Among all prenatally diagnosed cases, the mean gestational age at prenatal diagnosis was higher in cases with low maternal education than in cases with high maternal education (14.8 ± 3.6 vs. 14.0 ± 3.1 weeks, *p* = 0.03). No differences related to maternal geographic origin and occupation were observed.

Cases of TOPFA in all chromosomal anomalies were 1,051 corresponding to 74.1% of all cases and 88.6% of those prenatally diagnosed. Considering cases of all chromosomal anomalies prenatally diagnosed, we did not observe significant differences among classes of maternal education in the proportion of TOPFA (Table 4). The results were confirmed when only Italian mothers were considered. TOPFA did not differ among maternal occupation classes. We observed a significantly lower proportion of TOPFA for mothers from HMOC compared to the Italian ones (OR = 0.47, *p* = 0.003).

**Table 4:** Percentage and adjusted Odds Ratio of termination of pregnancies after prenatal diagnosis by maternal characteristics in prenatally diagnosed cases for all chromosomal anomalies and for Down syndrome.

	**Chromosomal anomalies**			**Down syndrome**		
	**%**	**OR^#^**	***P***	**%**	**OR^#^**	***P***
**Education**						
High	82.4	Ref.		85.6	Ref.	
Medium	86.4	1.58	0.062	93.2	3.24	0.005
Low	85.5	1.34	0.333	95.9	4.36	0.023
**Occupation**						
Manager/Professional	84.5	Ref.		87.0	Ref.	
Tech/Clerical/Service	90.2	1.70	0.101	96.2	3.89	0.009
Unskilled occupation/	86.1	1.11	0.797	90.3	1.44	0.542
Unemployed						
Housewives	79.4	0.98	0.965	82.4	0.76	0.719
**Geographic origin**						
Italian	90.4	Ref.		94.4	Ref.	
DC	86.4	0.60	0.415	92.3	0.66	0.698
HMOC	78.1	0.47	0.003	83.3	0.33	0.006

*OR, Odds Ratio*.

# *Adjusted by maternal age*.

For cases of Down syndrome, TOPFA were significantly more frequent in mothers with medium and low education (OR = 3.24, *p* = 0.005; OR = 4.36, *p* = 0.023, respectively). Considering only Italian mothers the results were confirmed (OR = 3.68, *p* = 0.004; OR = 10.49, *p* = 0.025, respectively). For mothers from HMOC, we observed a significantly lower proportion of TOPFA than for Italian mothers (OR = 0.33, *p* = 0.006).

## Discussion

In summary, using population-based data from a registry of congenital anomalies, we found that the probability of prenatal diagnosis for chromosomal anomalies was affected by different maternal sociodemographic characteristics. In particular, disparities persisted for women with lower education and for those from countries with a high migratory outflow. The findings referred to all chromosomal anomalies were largely confirmed for Down syndrome only, in agreement with the literature (3–5, 9, 18).

When data on income are not available, various factors have been used to reflect the concept of socioeconomic status. In our study, we have considered and analysed separately maternal education attainment and occupation. Maternal education was our main parameter and the choice was justified by its adequacy in evaluating information provided and in making an informed decision, besides being a good proxy of SES. It proved to be a reliable factor with the greatest impact on the outcome of interest, showing that women with a low level of education were less likely to have a prenatal diagnosis for chromosomal anomalies. Maternal geographic origin also highlighted differences, indicating that HMOC women, compared to the Italian ones, were less likely to have a prenatal diagnosis. These results could be the consequence of lesser access to the screening tests for Down syndrome observed in the same subgroups (i.e., women with low education level and women from HMOC) of the women residing in Tuscany (19).

These two features (education and geographic origin) were intermingled (46% of HMOC women were in the lower education group), but some specific explanations could be found for HMOC women. First, despite HMOC women being resident in Italy, the language barrier can be a key factor in disparities, resulting in lower health literacy (20, 21) and miscommunication between prenatal service providers and pregnant women (22). Second, according to a recent report on welfare and health in Tuscany (17), about 12% of foreign women would be late at the first visit during pregnancy (beyond the twelfth week of gestation), and 9% of them would make less than four visits during the whole period of pregnancy. These indicators are improving, compared to previous years, but are still at a sub-optimal level and could have an impact on the prenatal diagnosis. However, analysing only the cases from Italian mothers, differences were still present and, among mothers with lower education, the proportion of not prenatally diagnosed cases was higher with a borderline statistical significance (OR = 1.71, *p* = 0.058).

We also assessed maternal occupation which would seem to suggest that pregnant women in the lowest occupational subgroups were more likely not to have a prenatal diagnosis for chromosomal anomalies. Although these observations are to be considered with caution, due to the incompleteness of this variable, they still deserve to be taken into account both because they confirm the results of other studies (4, 5, 15) and because they represent a stimulus to improve data collection for future studies.

In Tuscany, the healthcare system is organized in three large areas (North-West, Centre, and South-East) with care services widespread in the territory, guaranteeing in this way homogeneous access to care. In each of these areas, there are large hospitals with specialized centres and universities. Furthermore, the Tuscany region, through a health performance assessment system, is committed to the continuous improvement of all the health services provided. In this context, the access to prenatal diagnosis is equally offered to all pregnant women residing in the region.

In 2006 the combined test (based on the combination of maternal age, foetal nuchal translucency thickness, maternal serum free β-human chorionic gonadotrophin and pregnancy-associated plasma protein-A) was introduced in Tuscany as a screening test for chromosomal anomalies and offered at a shared cost (the ultrasound scan was free, only the fee for the blood test was required). Over time, the screening test has become more widely used: in 2017, 75% of pregnant women had used the test. Thus, due to this important change in prenatal policy, we performed the analysis by sub-period. We found an increase of prenatal diagnoses (81.9% in 2005–2011 vs. 85.3% in 2012–2017), but differences among the sociodemographic groups were confirmed. In 2019, in Tuscany, the combined test was made free of charge and the NIPT (Non-invasive Prenatal Testing) for the analysis of foetal DNA was introduced. Therefore, it will be important in the future monitoring their impact on the population subgroups.

In our study population, a large proportion of women does not continue the pregnancy after a diagnosis of chromosomal congenital anomalies. Among cases prenatally diagnosed, we did not observe differences in the proportion of TOPFA for levels of maternal education. Conversely, for cases of Down syndrome only, we observed a higher proportion of TOPFA in mothers with low maternal education, in contrast with the less frequent prenatal diagnosis in the same subgroup. These findings confirm that the knowledge of a diagnosis in the prenatal period represents a fundamental element in the decision to continue or to terminate the pregnancy.

Regarding the maternal geographic origin, the proportion of TOPFA was lower in women from HMOC. This result suggests that a different cultural background could play a role in the decision to continue or terminate the pregnancy (23).

Other factors such as previous history termination of pregnancy, gestational age at prenatal diagnosis, number of children of the woman may influence the decision to terminate the pregnancy (24). We have added these variables in the statistical model and the differences in the proportion of TOPFA between the socio-demographic subgroups remained very similar. The results were confirmed also adjusting for gestational age at diagnosis although it was higher in cases with low maternal education.

Prenatal diagnosis has to be considered an important factor in informed decision making and it is recommended that all women, regardless their sociodemographic characteristics, have an equal likelihood to access the prenatal care services (18). Multiple factors influence the decision whether to continue or terminate the pregnancy (24, 25) and ultimately, the parents' decision, whatever it may be, must be respected. Language, cultural traditions and religious beliefs are important aspects that have an impact on the personal decision process. But the nature of this study does not allow us to evaluate them. Other studies including interviews of women following their decision to continue or terminate the pregnancy could provide helpful information and in-depth analysis of their role.

The main strength of the present study is that data are ascertained by a registry based on a regional network allowing the standardized collection of cases diagnosed and validated by health centres. Furthermore, as RTDC is a population-based registry, the cases of all the residing population were collected, reducing the selection bias. Moreover, results from a study using data at the population level represent the most accurate information to detect the presence of sources of health inequalities and, consequently, to help in addressing more efficient public health policy.

The main limitation of the study is the proportion of missing values, especially for maternal education (503 cases, corresponding to 35.5%) and maternal occupation (626, 44.1%). Twenty-eight of the 503 cases with no information on maternal education did not have a prenatal diagnosis (5.6%). However, among them, 61.1% comes from HMOC and we have observed that maternal education was lower in mothers from HMOC. Furthermore, we observed that 447 out of the 503 cases with unknown maternal education, were cases of TOPFA; in addition, we did not observe significant differences in the proportion of TOPFA among the subgroups in cases with known maternal education. Therefore, from these two pieces of information, it is reasonable that the unknown information on maternal education seems not to have generated a bias in our study. Regarding the maternal occupation, the proportion of missing values was even higher than maternal education. Another limitation of these data was the difficulty to define an accurate classification that can be used as a proxy of SES.

Although we have observed some differences in the sociodemographic subgroups suggesting conditions of inequalities, a more accurate and complete data collection on SES variables is needed to better detect possible conditions of disparities.

## Conclusions

In our study evidence of differences in the probability of prenatal diagnosis of chromosomal anomalies associated with maternal education and geographic origin was found. Our results suggest that, albeit the national healthcare system guarantees universal access to care services, disparities persist in the access to prenatal diagnosis due to socioeconomic status, in particular for women with lower education and women from countries with a high migratory outflow. These findings could give an insight into some aspects concerning the efficacy of the healthcare policies and can further stimulate the counselling activity for an informed choice. Besides, they should be monitored also in light of the recent availability of non-invasive screening tests. Population-based studies investigating sociodemographic disparities as well as healthcare practices can provide essential information for targeted public health programs.

## Data Availability Statement

The data analyzed in this study was subject to the following licenses/restrictions: the owner of data is Regione Toscana that might provide data upon request. Requests to access these datasets should be directed to www.regione.toscana.it.

## Author Contributions

MS conceived the study. MS, LM, and AC designed the study. MS and LM performed the data analysis, interpreted the results, and drafted the manuscript. SB, LP, and AP contributed to the design of the study and the interpretation of the results. All authors critically revised the manuscript and approved the final version.

## Conflict of Interest

The authors declare that the research was conducted in the absence of any commercial or financial relationships that could be construed as a potential conflict of interest.

## Abbreviations

RTDC: Registro Toscano dei Difetti Congeniti
EUROCAT: European network of population-based registries for the epidemiological surveillance of congenital anomalies
TOPFA: termination of pregnancy for foetal anomaly following prenatal diagnosis
HMOC: High Migratory Outflow Countries.
